# Effects of Nogo-A Silencing on TNF-*α* and IL-6 Secretion and TH Downregulation in Lipopolysaccharide-Stimulated PC12 Cells

**DOI:** 10.1155/2015/817914

**Published:** 2015-10-25

**Authors:** Jianbin Zhong, Shengnuo Fan, Zhenwen Yan, Songhua Xiao, Limei Wan, Chibang Chen, Simin Zhong, Lu Liu, Jun Liu

**Affiliations:** ^1^Neurology Department, People's Hospital of Zengcheng City (Boji Hospital Affiliated to Sun Yat-sen University), Guangzhou 511300, China; ^2^Neurology Department, Sun Yat-sen Memorial Hospital, Sun Yat-sen University, Guangzhou 510120, China

## Abstract

Parkinson's disease (PD) is a common degenerative disease that lacks efficient treatment. Myelin-associated neurite outgrowth inhibitor A (Nogo-A) is relevant with inhibition of nerve regeneration and may play vital role in pathogenesis of PD. The study aimed to establish the shRNA expression plasmids of Nogo-A gene and explore the regulatory effects of Nogo-A silencing on the expression of inflammation factor tumor necrosis factor-alpha (TNF-alpha) and interleukin-6 (IL-6) as well as tyrosine hydroxylase (TH) in lipopolysaccharide- (LPS-) stimulated rat PC12 cells. The results showed that both mRNA and protein levels of Nogo-A in pGenesil-nogoA-shRNA group were downregulated. The viabilities of PC12 cells decreased with increase of LPS concentrations. LPS significantly increased the supernatant TNF-alpha and IL-6 concentrations and reduced TH protein expression in PC12 cells, while silencing Nogo-A could block these effects. These results suggested that LPS can activate PC12 cells to secrete inflammatory cytokines and lower the TH expression, which can be regulated by Nogo-A gene silencing. Nogo-A silencing might provide new ideas for PD treatment in the future.

## 1. Introduction

Parkinson's disease (PD) is a degenerative disease of extrapyramidal system commonly seen in middle-aged and aged people with the main pathological manifestations including loss of substantia nigra dopaminergic neurons and formation of Lewy body (LB) in nerve cells. As an incurable progressive disease, PD is primarily currently treated symptomatically without significant efficacy. Recent studies have suggested that gene therapy is likely to be a radical strategy for many incurable diseases in the near future by finding and targeting disease-causing genes and pathways; therefore, searching for PD-related genes and applying gene therapy may be one of the most effective means to treat PD [1, 2]. Myelin-associated neurite outgrowth inhibitor (Nogo) is a myelin sheath-derived protein expressed in oligodendrocytes and extensive neurons and is associated with inhibition of neurite outgrowth of the central nervous system. There are three forms of Nogo, Nogo-A, Nogo-B, and Nogo-C. Nogo-A is a myelin-associated protein currently known to be most effective in central nervous system (CNS) inhibition. Its primary knowledge is mainly originated from the findings in experiments of central nerves repair after nerve injury. The results have indicated that Nogo-A, expressed by oligodendrocytes in both mature brain and spinal nerves, can inhibit neurite outgrowth and regeneration. Studies have shown that the inhibition effects of Nogo-A are involved in NgR signal transduction and RhoA. Researchers have found that neurons also express Nogo proteins, and the expression levels are increased in neuronal injury [3]. Both Nogo-A monoclonal antibody application and Nogo-A gene downregulation are able to partially counteract the inhibition of neurite regeneration and promote the regeneration of damaged nerves, thus enabling partial recovery of the neurological function. Neurite outgrowth inhibition by Nogo-A is also related with activation of the inflammatory cytokines secretion in neurons [4]. Nogo-A has been shown to be involved in Parkinson's disease [5]. Inflammatory cytokines such as TNF-alpha and IL-6 also play important roles in the occurrence and progression of PD [6]. However, whether Nogo-A regulates generation of the inflammatory factors, thus being involved in the occurrence and progression of PD, is still unknown. This study used lipopolysaccharide- (LPS-) stimulated PC12 cells to establish PD model and silenced Nogo-A genes using RNA interference technology and primarily explore the status of secretion of inflammatory cytokines TNF-*α* and IL-6 and tyrosine hydroxylase expression in the established model, thus providing new ideas for PD treatment.

## 2. Materials and Methods

### 2.1. Materials

*Cells and Main Reagents*. PC12 cells were provided by Professor Jun Liu. Lipopolysaccharide (LPS) and dimethyl methyl sulfate (DMSO) were purchased from Sigma Corporation. CCK-8 kits were purchased from Japan. Trypsin, DMEM, fetal bovine serum, penicillin, and streptomycin were purchased from Gibco Corporation. Silencer siRNA construction Kit was purchased from Ambion Company, plasmids were purchased from Shanghai GenePharma Company, Lipofeetamine2000 transfection reagents were purchased from Invitrogen Corporation, and TRIzol and SYBR Prime Script Real-Time Q-PCR kit were purchased from TAKARA Corporation. Anti-Nogo-A antibodies and anti-tubulin (*β*-tubulin) antibodies were purchased from Santa Cruz, USA. Anti-tyrosine hydroxylase antibodies and ECL kits were purchased from Merck Millipore. TNF-*α* and IL-6 ELISA kits were purchased from Shanghai Enzyme-linked Biotechnology Co., Ltd. Others were analytically pure reagents produced in China.

### 2.2. Methods

#### 2.2.1. Establishment of Nogo-A shRNA Expression Vector and PC12 Cells Transfection

Nogo-A mRNA sequences were retrieved from the GenBank (genes registration number: NM-031831.1), shRNA sequences were designed following the principle of siRNA design, Nogo-A gene was searched at 3075 bp after the start codon, the sequences with AA + N19 + UU were selected, and qualified 19 bp sequences were matched in the NCBI database using blast to search nucleotide sequence homology and ensure that the targeted gene is unique, thus determining the shRNA used in this research. Nonsilencing shRNA was designed as 19 bp double stranded RNA without homology to the rat gene sequences. ShRNA sense strand: 5′-AAAUCAGAUGAAGGCCACCCAUUTT-3′, antisense strand: 5′-AAUGGGUGGCCUUCAUCUGAUUU-3′, nonsilencing shRNA sense strand: 5′-AAAUGACUCAUUGGCGCCUCGUUTT-3′, and antisense strand: 5′AACGAGGCGCCAAUGAGUCAUUUTT-3′. Synthesized fragments were subcloned into pGenesil-1.1 to establish pGenesil-NogoA-shRNA, and pGenesil-1.1 was used as empty plasmid vector. Traditionally cultured PC12 cells were used as control group. Lipofectamine2000 was used to transfect PC12 cells, and the transfected cells were cultured for 48 h.

#### 2.2.2. Detection of Nogo-A Expression Using RT Q-PCR and Western Blot Assays

*Design and Synthesis of Nogo-A and β-Actin Primers*. Nogo-A upstream primer: 5′-CCTGCTCTCGGTGACTATCA-3′; Nogo-A downstream primer: 5′-GTAAACACCCACATCAACACT-3′; *β*-actin upstream primer: 5′-CTATCGGCAATGAGCGGTTCC-3′; *β*-actin downstream primer: 5′-TGTGTTGGCATAGAGGTCTTTACG-3′. Total RNA of the cells from three groups was extracted, cDNA synthesis was performed using reverse transcription Kit, and Nogo-A and *β*-actin primers were used for reaction using Bio-Rad Q-PCR instrument, thus obtaining the relative expression values of Nogo-A/*β*-actin.

*Detection of Nogo-A Protein Expression Using Western Blot Assays*. Total cellular proteins of the three groups were extracted using total protein extraction Kit. Proteins were transferred to nitrocellulose membranes by 10% and 5% SDS-PAGE electrophoresis, and the membranes were blocked by 10% defatted milk. Rabbit anti-Nogo-A antibodies (dilution ratio 1 : 1000) and anti-tubulin (*β*-tubulin) antibodies (dilution ratio 1 : 1000) were added to react at 4°C overnight, the membrane was rinsed by TBST 3 times and then added with horseradish peroxidase labeled goat anti-rabbit secondary antibodies (dilution ratio 1 : 5,000) to react at room temperature for 1 h, and ECL reagents were added to the membrane before exposure and photographing in dark room. Image analysis software Image J was used for quantitative analysis of the gray values of protein electrophoresis band.

#### 2.2.3. Establishment of Injury Model of LPS-Stimulated PC12 Cells

PC12 cells were seeded at 5 × 10^3^ cells/well in 96-well plate and treated with various concentrations of LPS (0.01 to 100 nmol/L), cultured for 24 h, added with 10 *μ*L CCK-8 reagents, and incubated for 1 h, and then OD450 of each well were determined using MICROPLATE READER to evaluate the cell vitality and determine the optimal concentration for cell injury. After the optimal concentration was evaluated, we further investigated whether silencing of Nogo-A would affect the viability of PC12 cells after LPS challenge.

#### 2.2.4. Detection of Supernatant TNF-*α* and IL-6 Using ELISA

According to the results of Section 2.2.3, the cells in control group, pGenesil-nogoA-shRNA group, and pGenesil-1.1 group were exposed to LPS at optimal concentration for 24 h, and TNF-*α* and IL-6 concentrations in supernatant fluid of each group were determined using conventional double antibody sandwich ELISA with microplate reader according to the ELISA Kit instructions.

#### 2.2.5. Detection of TH Expression Using Western Blot Assays

Based on the results of Section 2.2.3, the cells in control group, pGenesil-nogoA-shRNA group, and pGenesil-1.1 group were exposed to LPS at optimal concentration for 24 h, and Western blot assays were conducted using total protein extracts of each group according to the above described procedures.

#### 2.2.6. Statistical Analysis

Each experiment described above had been replicated at least 3 times independently. All experimental data were analyzed using SPSS17.0 software, and measurement data were expressed as mean ± standard deviation (*x* ± *s*) and analyzed using one-way ANOVA and LSD *t*-test. *p* < 0.05 indicated a statistically significant difference.

## 3. Results

### 3.1. mRNA and Protein Expressions in PC12 Cells after Nogo-A Silence

**Figure 1:**
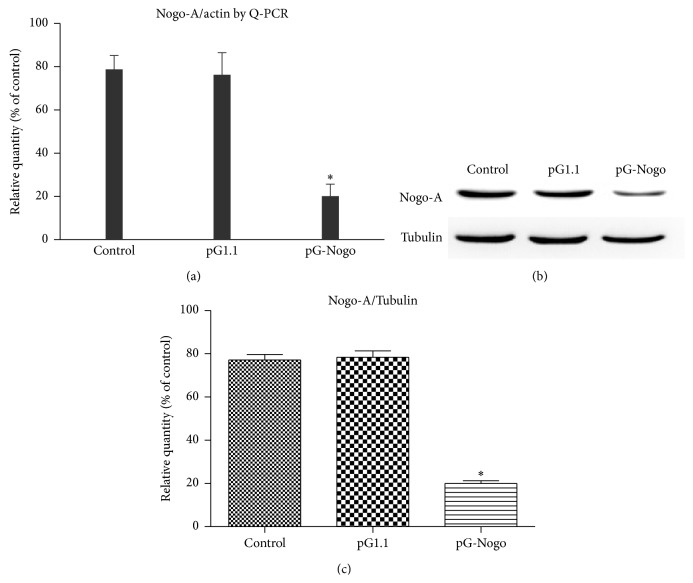
Detection of (a) Nogo-A mRNA levels using fluorescence quantitative PCR; (b, c) protein level using Western blot assays. pG1.1 refers to pGenesil-1.1 and pG-Nogo refers to pGenesil-nogoA-shRNA. ^*∗*^ *p* < 0.05.

RT Q-PCR results showed that control group and empty vector group were not significantly different in Nogo-A mRNA expression (*p* > 0.05), whereas pGenesil-nogoA-shRNA (pG-Nogo) group had a significantly lower Nogo-A mRNA expression than those in control group and empty vector group. Results of Western blot assays showed that, compared with the control group and empty vector group, Nogo-A proteins at hour 48 after shRNA were reduced with statistical significance (*p* < 0.05) (Figure 1).

### 3.2. Determination of Cell Viability

**Figure 2:**
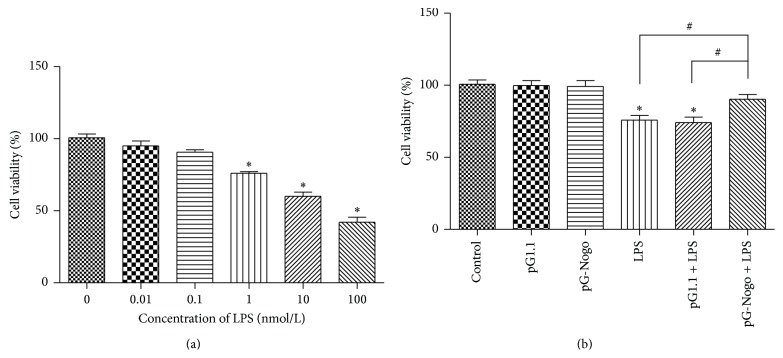
Cell viability tested by CCK8. (a) Gradual increase of LPS concentrations. (b) Silencing of Nogo-A on PC12 cells after treatment with LPS 1 nmol/L. ^*∗*#^ *p* < 0.05.

CCK8 tests showed that PC12 cell viabilities at hour 24 decreased gradually (*p* < 0.05) with the gradual increase of LPS concentrations (0.01 to 100 nmol/L, Figure 2(a)) and got a relatively ideal value at 1 nmol/L (76 ± 2.1%), which was used in subsequent experiments. Moreover, silencing of Nogo-A attenuated the viability loss of PC12 cells after LPS challenge (Figure 2(b)).

### 3.3. Detection of Supernatant TNF-*α* and IL-6 by ELISA

**Figure 3:**
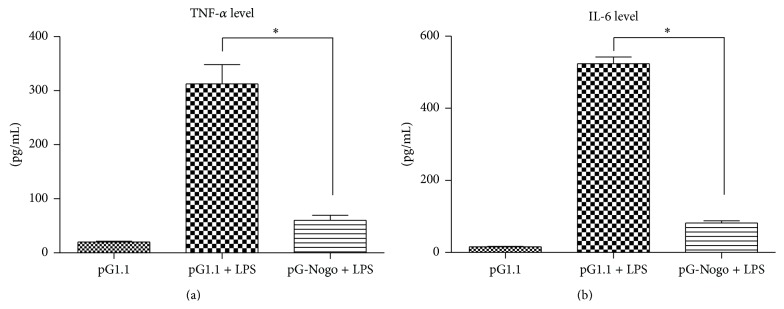
Detection of supernatant TNF-*α* and IL-6 using ELISA, ^*∗*^ *p* < 0.05.

LPS at work concentration of 1 nmol/L was used, control group (untreated PC12 cells), pGenesil-1.1 + LPS group, and pGenesil-nogoA-shRNA + LPS group were exposed for 24 h before the ELISA assays were performed and the OD450 were recorded using MICROPLATE READER, the standard values were calculated according to the standard curve, mean values of three independent experiments were taken (*p* < 0.05), and the results showed that LPS significantly increased the supernatant TNF-alpha and IL-6 concentrations, while silencing Nogo-A could block these effects (Figure 3).

### 3.4. Detection of TH and Nogo-A Expression Using Western Blot Assays

**Figure 4:**
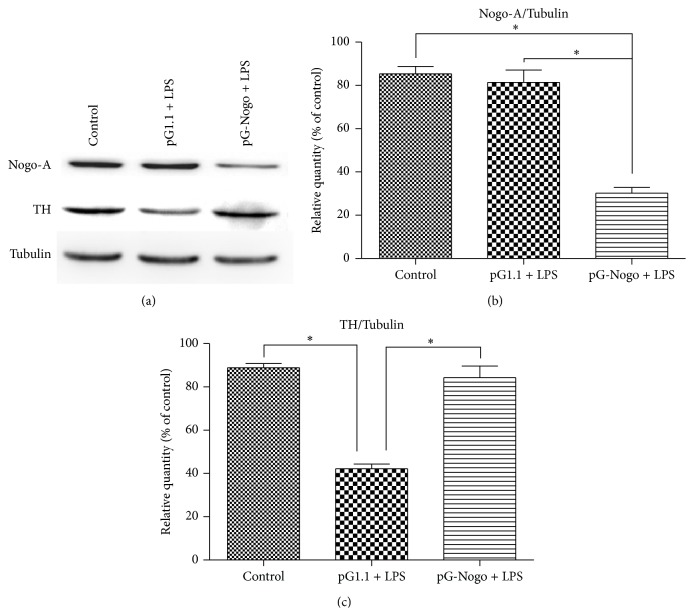
TH and Nogo-A levels of various groups determined by Western blot assays, ^*∗*^ *p* < 0.05.

LPS at work concentration of 1 nmol/L was used, control group (untreated PC12 cells), pGenesil-1.1 + LPS group, and pGenesil-nogoA-shRNA + LPS group were exposed for 24 h before total proteins were extracted and determined by Western blotting assays, and the results showed that LPS could significantly reduce TH protein expression in PC12 cells, while Nogo-A silencing somewhat restored the protein level compared with the group treated by LPS alone (Figure 4).

## 4. Discussion

Currently confirmed neurite regeneration inhibitory factors include three classes, namely, soluble myelin-associated glycoprotein (MAG), oligodendrocyte myelin glycoprotein (OMgp), and three of Nogo proteins [7]. There are three isomers of homologous Nogo: Nogo-A, Nogo-B, and Nogo-C. Nogo-A performs strong neurite outgrowth inhibition effects. Nogo-66 loop of Nogo-A is bound by its receptor NgR and transferred to p75/RhoA/ROCK to activate the cAMP and cause intracellular signal transduction, thereby leading to nerve outgrowth inhibition.

Nogo-A is mainly distributed in oligodendrocytes and additionally is highly expressed in the neurons. Wang et al. [8] found that Nogo-A and Nogo receptors are highly expressed in the neuron nucleus of the cerebral cortex, hippocampus, hypothalamus, globus pallidus, caudate-putamen nucleus, and substantia nigra. Studies found that the positive density of Nogo-A in anterior horn motor neurons of mouse spinal cord was very high. The distribution characteristic of Nogo-A determined that its abnormal expression played an important role in the occurrence and progression in nervous system diseases such as cerebral and spinal trauma, stroke, and neurodegenerative diseases [9, 10]. As a common degenerative disease of the nervous system, PD is related with the loss and regeneration inhibition of dopaminergic neuron, and Nogo-A had been confirmed to be involved in the process [11–13].

Therefore, as a key factor in development and progression of nervous system diseases described above, Nogo-A may serve as potential therapeutic targets of various diseases, especially the PD. Chen et al. [14] found that application of specific antibody of Nogo-A protein IN-1 could block the neurite inhibition activity of Nogo-A, and GrandPré et al. [15] found that NgR antagonists could facilitate the neurite regeneration. Yang et al. [16] found that Nogo-A silencing could promote the recovery of demyelinating disease. Wang et al. [17] found that neurite outgrowth inhibition by Nogo-A was closely related with Wnt pathways, c-Jun, and c-Myc. Some scholars believed that Nogo-A is also involved in neuronal differentiation, apoptosis, and generation of free radicals [18, 19]. Interestingly, studies of Schapira et al. [2] indicated that neural inhibition of Nogo-A was related with inflammatory mediators.

With reference to various investigations [20, 21], we established PD model using LPS-stimulated PC12 cells, and the results showed that LPS significantly increased the supernatant TNF-alpha and IL-6 concentrations of PC12 cells while inhibiting the PC12 cell vitality, indicating that inflammation factor played an important role in PD pathogenesis. Nogo-A silencing led to significant decline of inflammation factors secretion and alleviated the cell viability loss induced by LPS, suggesting that Nogo-A gene was involved in inflammation factor secretion in PD. As an enzyme responsible for catalyzing conversion of amino acid l-tyrosine to dihydroxyphenylalanine, TH plays a decisive role in the synthesis of dopamine. Sufficient TH expression can lead to dopamine synthesis reduction and eventual PD pathogenesis. Therefore, it has been recognized as an indicator of PD [1, 22, 23]. The results of this study showed that LPS could significantly reduce TH protein expression in PC12 cells. TH protein expression in group of Nogo-A silencing was somewhat restored compared with the group treated by LPS alone, showing that Nogo-A influenced the TH expression. These results suggest that Nogo-A has promotive effects on PD and may accelerate the process of PD possibly by increasing the inflammatory substances secretion and reducing the TH expression through a certain way, but the exact mechanism is unclear and needs further investigation in the future.

## 5. Conclusion

This study indicated that Nogo-A had promotive effects on PD and might accelerate the process of PD possibly by increasing TNF-alpha and IL-6 secretion and reducing the TH expression. Silencing the Nogo-A presented a protective effect in the study, which provided new ideas for PD treatment.
